# 

**Published:** 1940

**Authors:** 


### CONTENTS.

| | Page |
|---|---|
| The Twenty-Ninth Long Fox Memorial Lecture: Popular Misconceptions in Medical Matters. By Prof. T. B. Davie, M.D., F.R.C.P. | 107 |
| Midwifery in Bristol. By H. J. Drew Smythe, M.D., M.S., F.R.C.S., F.R.C.O.G., and H. L. Shepherd, M.B., Ch.M., F.R.C.O.G | 117 |
| Induction of Labour by High Puncture of the Membranes. By Coralie Rendle Short, M.B., Ch.B., M.R.C.O.G. | 121 |
| The Bombing of Bristol | 128 |
| The Shelter Problem | 130 |
| Reviews of Books | 133 |
| Editorial Notes | 141 |
| Obituary: Norman Francis Clifford Burgess, M.A., M.D., M.R.C.P., Lieutenant-Colonel R.A.M.C.(T.) | 142 |
| Meetings of Societies | 143 |
| The Medical Library of the University of Bristol | 144 |
| Publications Received | 145 |
| Local Medical Notes | 146 |

